# The K_ATP_ channel in migraine pathophysiology: a novel therapeutic target for migraine

**DOI:** 10.1186/s10194-017-0800-8

**Published:** 2017-08-23

**Authors:** Mohammad Al-Mahdi Al-Karagholi, Jakob Møller Hansen, Johanne Severinsen, Inger Jansen-Olesen, Messoud Ashina

**Affiliations:** 1 Danish Headache Center, Department of Neurology, Rigshospitalet Glostrup, Faculty of Health and Medical Sciences, University of Copenhagen, Nordre Ringvej 57, DK-2600 Copenhagen, Denmark; 2 Danish Headache Center, Department of Neurology, Glostrup Research Park, Rigshospitalet Glostrup, Copenhagen, Denmark

**Keywords:** Migraine, K_ATP_ channel, K_ATP_ channels, Headache, Levcromakalim, Cromakalim

## Abstract

**Background:**

To review the distribution and function of K_ATP_ channels, describe the use of K_ATP_ channels openers in clinical trials and make the case that these channels may play a role in headache and migraine.

**Discussion:**

K_ATP_ channels are widely present in the trigeminovascular system and play an important role in the regulation of tone in cerebral and meningeal arteries. Clinical trials using synthetic K_ATP_ channel openers report headache as a prevalent-side effect in non-migraine sufferers, indicating that K_ATP_ channel opening may cause headache, possibly due to vascular mechanisms. Whether K_ATP_ channel openers can provoke migraine in migraine sufferers is not known.

**Conclusion:**

We suggest that K_ATP_ channels may play an important role in migraine pathogenesis and could be a potential novel therapeutic anti-migraine target.

## Introduction

Adenosine 5′-triphosphate-sensitive K^+^ (K_ATP_) channel openers have been used in clinical trials for the treatment of hypertension and asthma. The most common side effect mentioned during treatment with K_ATP_ channel openers was headache (62, 64, 66–79) (Tables 2 and 3). However, only little attention has been focused on the role of K_ATP_ channels in migraine pathophysiology.

K_ATP_ channels were originally identified in cardiomyocytes [1], but have also been found in several tissues, including pancreatic α- and ß-cells, smooth muscle, skeletal muscle and central neurons [2, 3]. The channels belong to the family of inwardly rectifying K^+^ channels that are inhibited at physiological intracellular levels ATP/ADP ratio. When intracellular ATP is reduced under conditions of metabolic challenges they open. K_ATP_ channels are critical in regulating insulin secretion, controlling vascular tone, and protecting cells against metabolic stress [2, 4, 5].

Over the past three decades, some preclinical evidence has emerged indicating that K_ATP_ channels may play an important role in migraine pathophysiology. In particular, the vasodilation effect of K_ATP_ channels is relevant, since it is has been established that endogenous neurotransmitters that trigger migraine attacks are often associated with dilation of cranial arteries [6].

Here we review preclinical and clinical studies on K_ATP_ channels and discuss the K_ATP_ channel as a novel therapeutic target for migraine treatment.

## Molecular structure and isoforms

The K_ATP_ channel is a hetero-octameric complex that consists of four pore-forming K^+^ inwardly rectifying (Kir) subunits and four regulatory sulfonylurea receptor (SUR) subunits [7].

The Kir6.x subunit exists in two isoforms, Kir6.1 and Kir6.2. The SUR subunit belongs to the ATP-binding cassette (ABC) transporter family, regulated by sulfonylurea, with three isoforms, SUR1, SUR2A, and SUR2B [7, 8].

**Fig. 1:**
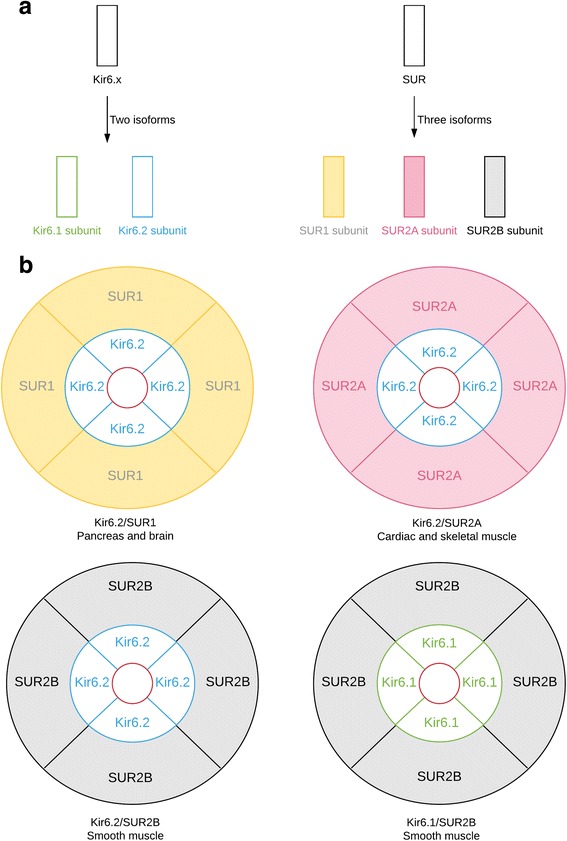
Molecular structure and isoforms. **a** Two major Kir6.x isoforms (Kir6.1 and Kir 6.2) and three major SUR isoforms (SUR1, SUR2A and SUR 2B) have been identified. **b** Kir.x subunits combine tissue-specifically with different SUR subunits to form various native K_ATP_ channels. Pancreatic, cardiac and smooth muscle K_ATP_ channels are made up of Kir6.2/SUR1, Kir6.2/SUR2A and Kir6.1 (or Kir6.2)/SUR2B, respectively [2]. Kir, inwardly rectifying K^+^ channels; SUR, sulfonylurea receptor

**Fig. 2:**
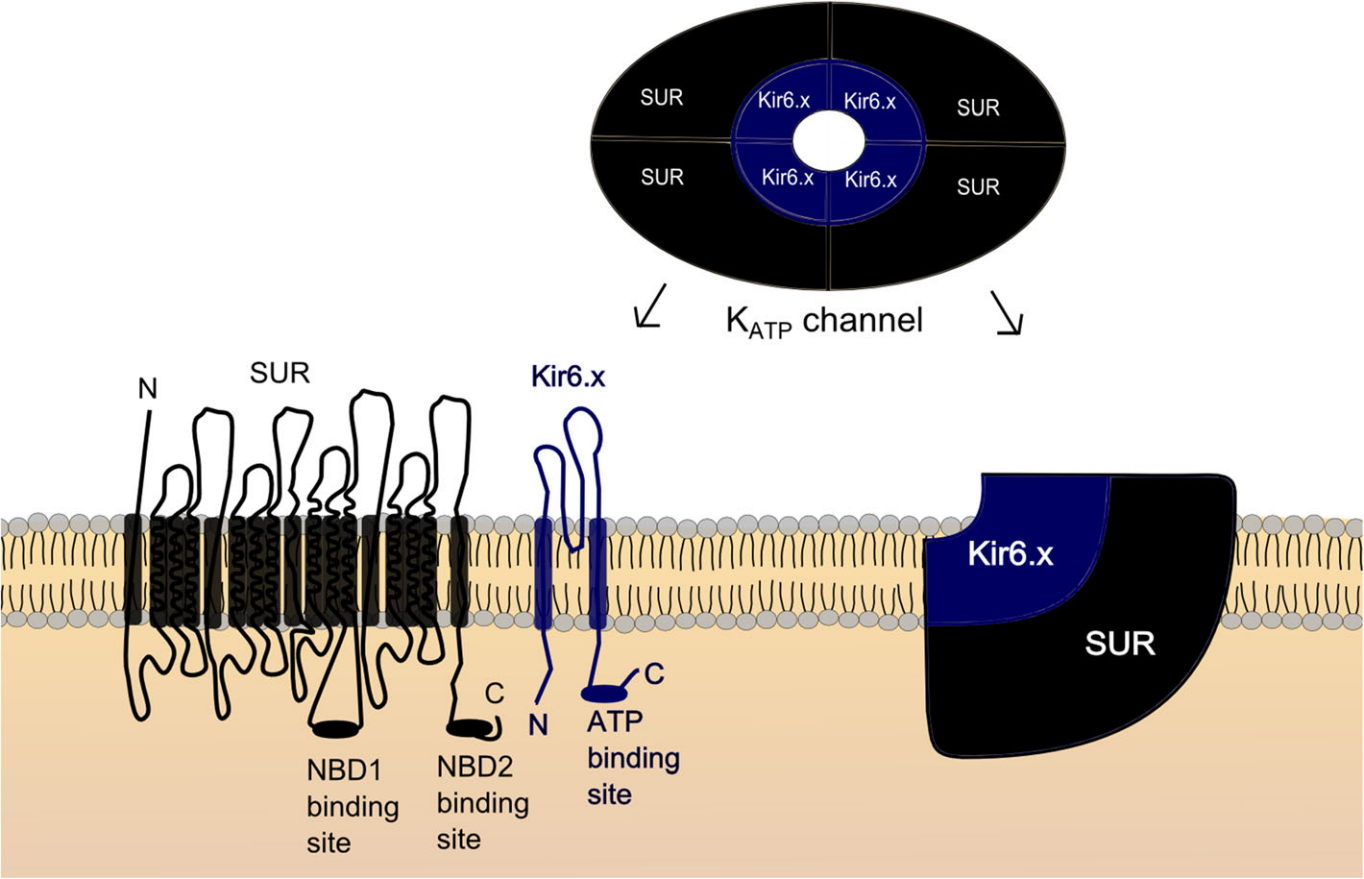
Schematic diagram of the K_ATP_ channel. Kir6.x subunits have two transmembrane domains, and a large cytoplasmic domain including an inhibitory binding site for ATP [8, 84]. SUR subunits have many transmembrane domains and two intracellular nucleotide binding domains (NBD1 and NBD2), which stimulate opening of the channel after binding to MgADP [85]

K_ATP_channels have specific tissue expression with different compositions of Kir6.x and SUR subunits which lead to distinct functional properties (Figs. 1 and 2 and Table 1).

**Table 1:** Distribution of K_ATP_ channels

Subtypes of K_ATP_channels	Tissue expression	Migraine related structures
Kir6.2/SUR1	Pancreas and brain	DRG, TG and TNC from rats (20–24, 26).
Kir6.2/SUR2A	Cardiac and skeletal muscle	
Kir6.2/SUR2B	Smooth muscle	DRG, TG, TNC, BA and MCA from rats(20–24, 26).
Kir6.1/SUR2B	Smooth muscle	MMA from rats, pigs and human; MCA from rats and pigs; BA, DRG, TG and TNC from rats (20–24, 26).

*DRG* Dorsal root ganglia, *TG* trigeminal ganglion, *TNC* trigeminal nucleus caudatus, *BA* basilar artery, *MMA* middle meningeal artery, *MCA* middle cerebral artery

## Channel function

**Fig. 3:**
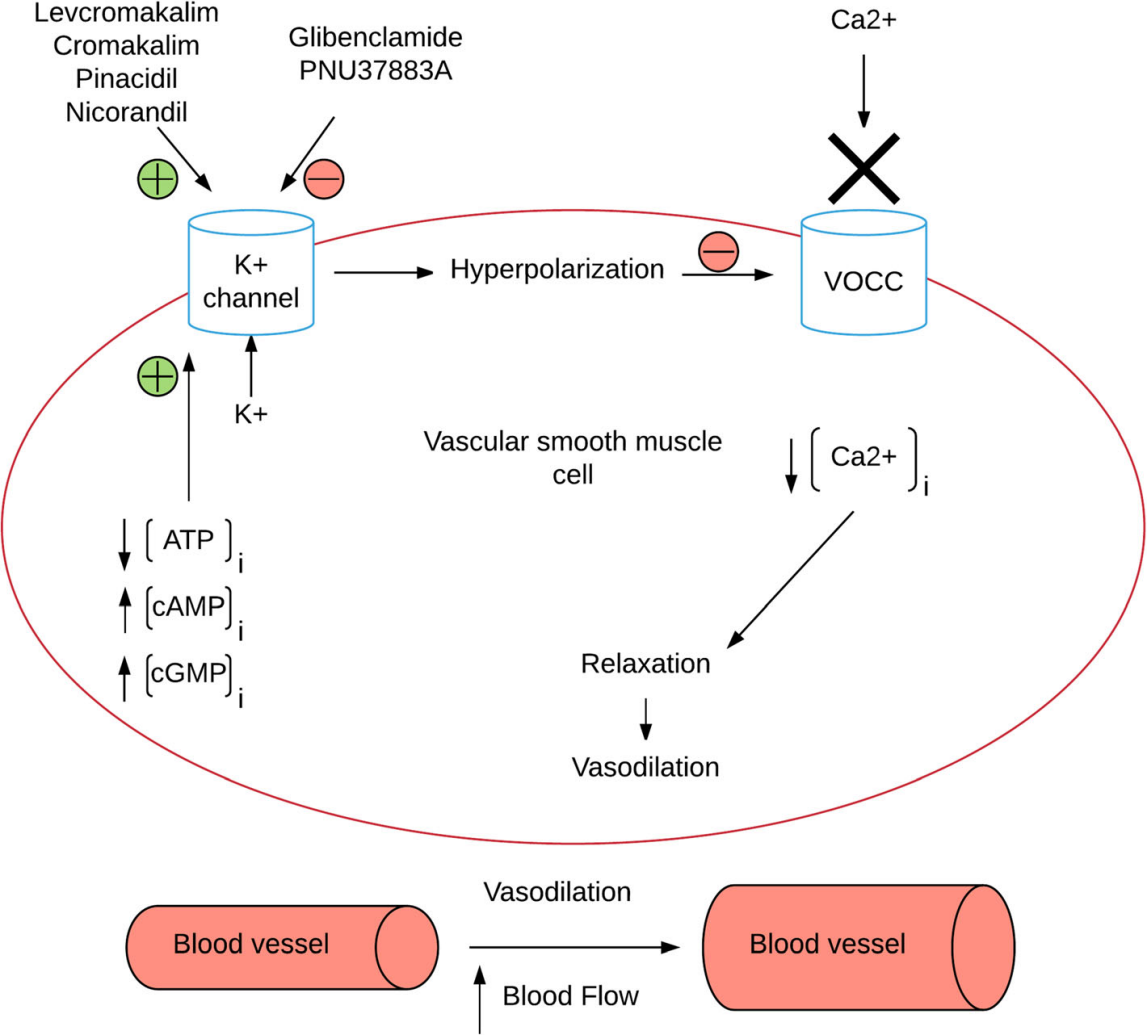
Opening of vascular ATP sensitive K channels. Endogenous molecules (ATP, cAMP and cGMP) and exogenous pharmacological agents (cromakalim and glibenclamide) regulate the activity of K_ATP_ channels, which help controlling the vascular tone

K_ATP_ channel activity is controlled by changes in concentrations of intracellular ATP and magnesium adenosine diphosphate (Mg-ADP). K_ATP_ channels couple the metabolic state of the cell to the membrane potential and thus play a crucial role in many tissues under both physiological and pathological conditions [9]. K^+^ channels participate in the regulation of vascular tone, including cerebral arteries [10]. When intracellular ATP is reduced, K_ATP_ channels become activated; K^+^ efflux hyperpolarize the membrane and close voltage-operated Ca^2+^-channels (VOCC). The result is a decrease in cytosolic Ca^2+^ concentration followed by relaxation of vascular smooth muscle cells and an increase in blood flow [11]. The same applies if cells are exposed to metabolic stress such as ischemia or hypoglycemia [12]. Closure of K^+^ channels leads to membrane depolarization and constriction of the vessels [11]. In addition an increase in intracellular cAMP and cGMP levels activate K_ATP_ channels to produce vasodilation [11]. Synthetic K_ATP_ channel openers (like levcromakalim and cromakalim) and blockers (like glibenclamide, second generation of sulfonylurea and PNU37883A) directly activate or inhibit the vascular K_ATP_ channels, respectively [9] (Fig. 3).

## Distribution of K_ATP_ channels in migraine related structures

### Intracranial arteries

K_ATP_ channels are present and functional in intracranial arteries [13–15]. They are found in vascular smooth muscle cells and vascular endothelial cells [16, 17]. In rat cerebral arteries, the distribution of K_ATP_ channels varies with vessel size and brain region [18]. Real time polymerase chain reaction (RT-PCR) analysis revealed Kir6.1 and SUR2B subunits in middle meningeal artery (MMA) and middle cerebral artery (MCA) in rats and pigs [19, 20]. This profile of K_ATP_ channels is also identified in human MMA [21] (Table 1).

### Trigeminal ganglion and trigeminal nucleus caudalis

Kir6.1, Kir6.2, SUR1 and SUR2 are expressed in the trigeminal ganglion and trigeminal nucleus caudalis [22] (Table 1). In trigeminal neurons Kir 6.1 and Kir 6.2 immunoreactivity were expressed in cells with all soma sizes in all three divisions of the trigeminal ganglion [23].

## K_ATP_ channels openers and migraine signaling pathways

A number of endogenous vasoactive signaling molecules have been implicated in migraine [6], and K_ATP_ channels may interact with these molecules.

### Nitric oxide (NO)

In humans, infusion of the NO donor, glyceryl trinitrate, and inhibition of the breakdown of cGMP by sildenafil [24] provoke migraine attacks in migraineurs [25–27]. The NO-cGMP signaling pathway is involved in the relaxation of vascular smooth muscle [28]. In vitro studies with cerebral arteries isolated from rat and piglet and extra-cerebral arteries from rabbit reported that activation (opening) of K_ATP_ channels contributed to both cAMP- and cGMP-mediated vasodilation [29–31]. Yuan et al. [32] reported that sildenafil-induced vasodilation in porcine retinal arterioles was significantly inhibited by glibenclamide and suggested that cGMP signaling triggers opening of K_ATP_ channels. In contrast, NO-induced dural and pial artery dilation in rats was not attenuated by the K_ATP_ channel blocker, glibenclamide [33]. Together, these data suggest that interspecies differences are likely to explain the discrepancy in findings of the role of K_ATP_ channels in NO-induced vasodilation.

### Calcitonin gene-related peptide (CGRP)

**Fig. 4:**
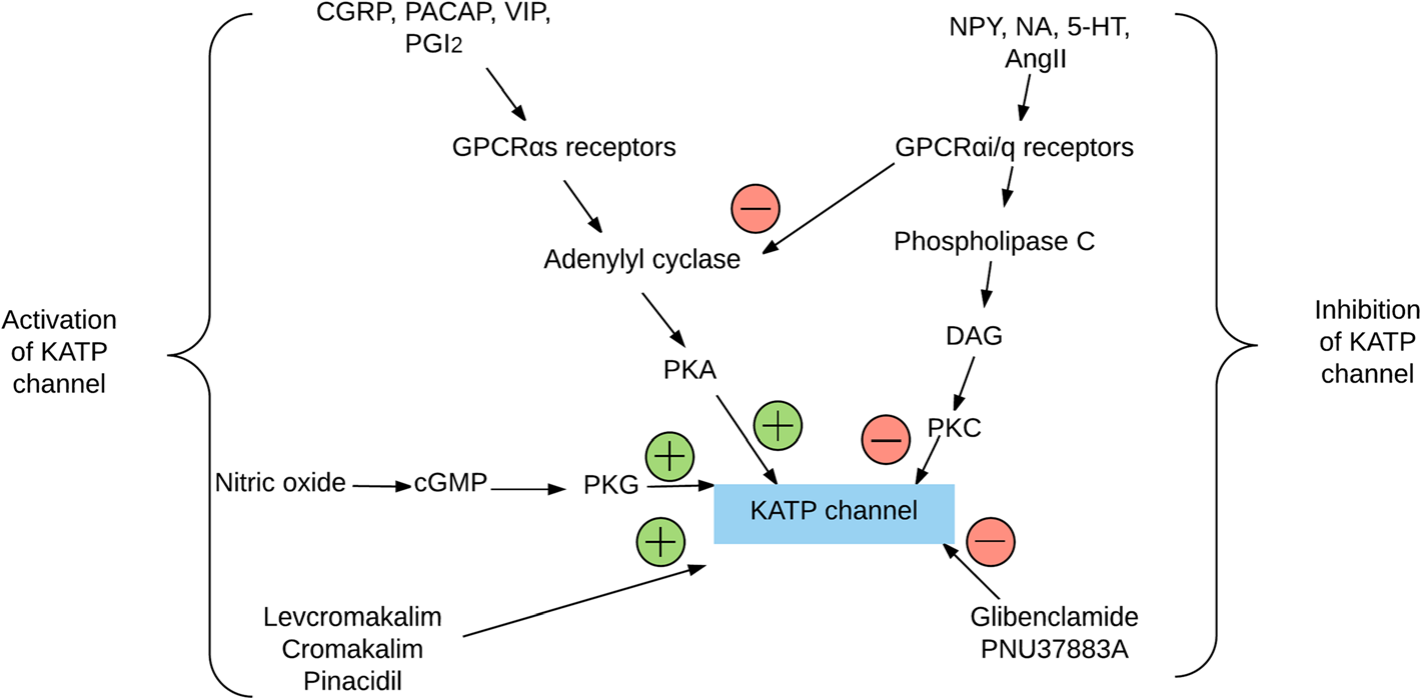
Signaling pathways through vascular smooth muscle K_ATP_ channels. Numerous endogenous vasodilators activate vascular smooth muscle K_ATP_ channels through adenylate cyclase and PKA phosphorylation. Conversely, endogenous vasoconstrictors inhibit vascular smooth muscle K_ATP_ channels through DAG and PKC phosphorylation. CGRP, calcitonin gene-related peptide; PGI_2_, prostaglandin I_2_; VIP, vasoactive intestinal peptide; AngII, angiotensin II; NPY, neuropeptide Y; NA, noradrenaline; 5-HT, 5-hydroxytryptamine; Gs, G-protein-coupled receptor alpha stimulation; Gi, G-protein-coupled receptor alpha i/q; DAG, diacylglycerol; PKA and PKC, protein kinase A and C, respectively

CGRP is one of the most potent endogenous vasodilators and major arteries in the intracranial circulation of man and animals are innervated by CGRP-containing nerve fibers [34–36]. Efficacy of CGRP antagonism is established in acute [37, 38] and preventive treatment of migraine [39]. CGRP activates vascular smooth muscle K_ATP_ channels indirectly through adenylate cyclase and protein kinase A (PKA) phosphorylation (Fig. 4) [40–43]. In rats, CGRP-induced dilation of the dural and pial arteries in vivo was shown to be inhibited by glibenclamide [33], but K_ATP_ channel openers do not interact with CGRP release in trigeminal ganglion and trigeminal nucleus caudalis [22]. This suggests that K_ATP_ channels are involved in CGRP-induced intracranial vasodilation.

### Pituitary adenylate cyclase activating polypeptide (PACAP)

Pituitary adenylate cyclase activating polypeptide (PACAP) is a potent endothelium independent vasodilator of various vascular beds, including cerebral arteries [44, 45]. In vivo and in vitro studies have demonstrated that PACAP dilates cranial arteries in different species, e.g. human cerebral arteries [34, 46, 47], pig pia artery, canine basilar artery, cat cerebral arteries, rabbit posterior cerebral arteries and rat middle cerebral arteries [48–52]. Emerging data suggest that PACAP or it receptors are a promising target for migraine therapeutics [53]. PACAP has three types of receptors; Pituitary adenylate cyclase PAC_1_ (*p*ituitary *a*denylate *c*yclase receptor 1), VPAC_1_ (*v*asoactive intestinal peptide and *p*ituitary *a*denylate *c*yclase receptor 1) and VPAC_2_ (*v*asoactive intestinal peptide and *p*ituitary *a*denylate *c*yclase receptor 2) [54] the two latter ones are also activated by vasoactive intestinal peptide and all three receptors are found in cerebral artery smooth muscle cells [55]. Through these receptors, PACAP leads to an increase in intracellular cAMP, which activates PKA and produces vasodilation by several mechanisms including activation of K_ATP_ channels (Fig. 4) [45]. Interestingly, glibenclamide could partially inhibit PACAP induced vasodilation in cerebral, coronary and pulmonary arteries, suggesting that PACAP may also activate K_ATP_ channels [44, 45].

### Prostaglandins

Prostacyclin (PGI_2_) activates and sensitizes meningeal sensory afferents, and provokes immediate migraine-like attacks in migraine sufferers [56]. PGI_2_ also increases K_ATP_ channel activity in vascular smooth muscle preparations by cAMP-dependent PKA activation [57] (Fig. 4).

## Headache induced by K_ATP_ channels openers

In the late 80’s there was a tremendous interest in developing novel K_ATP_ channel openers for hypertension, angina pectoris and asthma. Three pharmacological drugs were developed, pinacidil, nicorandil and levcromakalim. One of most common adverse events after treatment reported in these studies was headache [58–63].

Six clinical trials with pinacidil have been published for treatment of essential hypertension. Between 7% and 21% of the patients reported headache as an adverse effect (Table 2).

**Table 2:** Headache incidences registered during randomized controlled trials (RCT) and open label clinical trials with pinacidil

Paper	Study design	Dose (daily)	Indication	No. of patients	Headache No.
Muiesan et al. 1985, Eur. J. Clin. Pharmacol [86].	RCT	30–75 mg	Essential hypertension	30	2 (7%)
Laher & Hickey 1985, J. Int. Med. Res [87].	Open label	12.5 mg	Healthy volunteers	12	1 (8%)
D’Arcy et al. 1985, Eur. J. Clin. Pharmacol [88].	Open label	20–100 mg	Essential hypertension	23	4 (17%)
Zachariah et al. 1986, Eur. J. Clin. Pharmacol [89].	RCT	62 mg (mean)	Essential hypertension	23	----
Sterndorff & Johansen 1988, Acta Med. Scand [90].	RCT	25–100 mg	Essential hypertension	71	7 (10%)
Goldberg 1988, J. Cardiovasc. Pharmacol [91].	RCT	25–100 mg	Essential hypertension	145	31 (21%)

Nicorandil was tested for the treatment of angina pectoris and ischemic heart disease. 23% to 88% of the patients reported headache as an adverse event (Table 3). The high incidence of headache is likely due to the mixed K_ATP_ channel opener and NO donor properties of nicorandil which thus cause vasodilation via two separate mechanisms.

**Table 3:** Headache incidences registered during randomized controlled trials (RCT) and open label clinical trials with nicorandil

Paper	Study design	Dose (daily)	Indication	No. of patients	Headache No.
Camm & Maltz, 1989, Am. J. Cardiol [92].	RCT	20–60 mg	Angina pectoris	8	20 mg 50% 40 mg 88% 60 mg 67%
Raftery et al. 1993, Eur. Heart Journal [93].	RCT	20 mg and 40 mg	Angina pectoris	18	11 (61%)
Roland 1993, Eur. Heart Journal [94].	Review	10–80 mg	Angina pectoris	1680	36%
Wolf et al. 1993, Eur.J.Clin.Pharmacol [95].	RCT	20–200 μg i.v.	Healthy volunteers	48	19 (40%)
Witchitz & Darmaon, 1995, Cardiovasc. Drugs& Therap [96].	Open label	20–40 mg	Angina pectoris	197	45 (23%)
Dunn et al. 1999, Pharmacoepidemiology and Drug safety [97].	Prescription-event monitoring (PEM) study	Varying	Angina pectoris & ischemic heart disease	13,260	477 (4%)

Levcromakalim was investigated for the treatment of asthma and essential hypertension. In these studies between 29% and 76% of the patients reported headache as an adverse event (Table 4).

**Table 4:** Headache incidences registered during randomized controlled trials (RCT) and open label clinical trials with levcromakalim

Paper	Study design	Dose (daily)	Indication	No. of patients	Headache No.
Singer et al. 1989, J. Hypertens [98].	RCT	1.5 mg	Essential hypertension	8	4 (50%)
Williams et al. 1990, Lancet [60].	RCT	1.5 mg	Asthma	16	10 (62%)
Kidney et al. 1993, Thorax [62].	RCT	0.125–0.5 mg	Asthma	25	19 (76%)
Suzuki et al. 1995, Arzneim.-Forsch./Drug Res [99].	Open label	0.5–1.0 mg	Essential hypertension	14	4 (29%)

The selective synthetic K_ATP_ channel openers levcromakalim and pinacidil have been shown to induce dilation in rat cranial arteries [13, 15, 19] and in isolated human cerebral arteries [64]. Moreover, the arterial dilation can be inhibited by synthetic K_ATP_ channel blockers like glibenclamide [10, 33] and PNU37883A [21, 65] (Fig. 3). These findings suggest that high incidences of headache could be due to vasoactive effect of the K_ATP_ channel openers in pain-sensitive extra- and/or intracerebral arteries.

## Discussion and future perspectives

K_ATP_channels are expressed in migraine-related structures such as the cranial arteries, TG and TNC [18–22, 66]. K_ATP_ channels are also connected to a number of key molecules in migraine pathogenesis, particularly nitric oxide, CGRP, PACAP and PGI_2_ known to provoke migraine attacks [56, 67–71]. Therefore, the K_ATP_ channels are interesting in migraine context.

Human experimental models have demonstrated that the activation of the cAMP and cGMP pathways can trigger headache in healthy volunteers and migraine attacks in migraine sufferers [6, 71, 72]. The cAMP and cGMP signaling pathways are crucial in the activation of K_ATP_ channels, which result in the relaxation of smooth muscle [29–31]. Furthermore, synthetic K_ATP_ channel openers like levcromakalim and pinacidil trigger headache in non-migraine patients [58–63]. Although a detailed description of levcromakalim- and pinacidil-induced headache and accompanying symptoms are lacking, these data support a role of K_ATP_ channels in migraine headache. Because K_ATP_ channel openers were tested for other indications, there are no available data on the potential migraine-inducing effects of pinacidil and levcromakalim in migraine patients. It is conceivable that both headache and migraine are underreported as adverse events, as was found for the phosphodiesterase inhibitors, cilostazol and sildenafil [73, 74].

In addition to the vasoactive effects, the K_ATP_ channels might also tap into other parts of the migraine cascade. For a number of patients, migraine attacks are associated with transient focal neurological symptoms called the aura [75], possibly caused by cortical spread depression (CSD) [76]. During CSD K^+^ conductance is increased, and CSD may be inhibited by Kir antagonist [77]. The fact that K_ATP_ channels open under cellular stress, as seen during long lasting depolarizations, could provide a link between K_ATP_ channels, CSD and migraine aura.

With regard to the migraine pain, it is worth noting that K_ATP_ channels are also found in peripheral nociceptive fibers [78] and activation of these channels play a crucial role in anti-nociception at both spinal and supra-spinal levels [23, 79]. The exact role of these findings in the headache induced by K_ATP_ channel openers is unknown.

If K_ATP_ channel openers are in fact able to trigger migraine, the next step to consider is whether K_ATP_ channel antagonists can relieve migraine. K_ATP_ blockers for the treatment of migraine should be selective for the Kir6.1/SUR2B subtype because of its dominant presence in vascular tissue (Table 1). The necessity of a subtype specific blocker is unavoidable because of occurrence of different subtypes in different tissues. Glibenclamide cannot be used due to its high affinity to the Kir6.2/SUR1 subtype of K_ATP_ channels present in the pancreas with hypoglycemia as a side effect [80]. PNU-37883A is a Kir6.1 selective K_ATP_ channel blocker that was originally developed as a diuretic drug [81, 82]. The drug was not approved to human studies because of its cardiac depressant activity in animal studies [83]. This precludes further clinical development of PNU-37883A due to possible serious adverse events but may not exclude further investigations in other blockers against Kir6.1 subunit because it is not clear if all blockers against Kir6.1 subunit have non-favorable effects. These findings indicate that the SUR2B subunit and the Kir6.1 subunit should be a potential target for the treatment of migraine, but proof of concept studies are needed to examine this hypothesis.

## Conclusion

Emerging evidence suggests that K_ATP_ channels could be involved in the pathophysiology of migraine. K_ATP_ channels exist in structures which are believed to be linked to the pathophysiology of migraine, including cerebral and meningeal arteries and the trigeminal system [19–22]. It is established that the cAMP signaling pathway and possibly cGMP signaling pathway are involved in the activation of K_ATP_ channels [29–31]. This is interesting in migraine contexts, as the two signaling pathways are likely to be crucial in the development of a migraine attack.

We suggest that the presented clinical and theoretical evidence support further studies of K_ATP_ channel openers in migraine context. Future human studies will help clarify the role of K_ATP_ channels in the pathophysiology of migraine.

## Abbreviations

ABC transporter: ATP-binding cassette transporter
BA: Basilar artery
CGRP: Calcitonin gene-related peptide
CSD: Cortical spread depression
DRG: Dorsal root ganglia
K_ATP_ channel: Adenosine 5′-triphosphate-sensitive K^+^ channel
Kir: K^+^ inwardly rectifying
MCA: Middle cerebral artery
Mg-ADP: Magnesium adenosine diphosphate
MMA: Middle meningeal artery
NBD: Nucleotide binding domains
NO: Nitric oxide
PACAP: Pituitary adenylate cyclase activating polypeptide
PGI_2_: Prostacyclin
SUR: Sulfonylurea receptor
TG: Trigeminal ganglion,
TNC: Trigeminal nucleus caudatus
VOCC: Voltage-operated Ca^2+^-channels
